# A prospective, randomized, single-blinded study comparing the efficacy and safety of dexmedetomidine and propofol for sedation during endoscopic retrograde cholangiopancreatography

**DOI:** 10.1186/s12871-024-02572-z

**Published:** 2024-05-28

**Authors:** Wenyou Zhang, Liangrong Wang, Na Zhu, Wenzhi Wu, Haiyan Liu

**Affiliations:** 1 Department of Anesthesiology, The First Affiliated Hospital of Wenzhou Medical University, Ouhai District, Shangcai Village, Nanbaixiang Town, Wenzhou, 325000 China; 2 Department of Gastroenterology, The First Affiliated Hospital of Wenzhou Medical University, Wenzhou, 325000 China

**Keywords:** Dexmedetomidine, Endoscopic retrograde cholangiopancreatography, Propofol, Sedation-related adverse events

## Abstract

**Background:**

Balanced propofol sedation is extensively used in endoscopic retrograde cholangiopancreatography (ERCP), but sedation-related adverse events (SRAEs) are common. In various clinical settings, the combination of dexmedetomidine with opioids and benzodiazepines has provided effective sedation with increased safety. The aim of this investigation was to compare the efficacy and safety of dexmedetomidine and propofol for sedation during ERCP.

**Methods:**

Forty-one patients were randomly divided into two groups: the dexmedetomidine (DEX) group and the propofol (PRO) group. Patients in the DEX group received an additional bolus of 0.6 μg kg^−1^ dexmedetomidine followed by a dexmedetomidine infusion at 1.2 μg kg^−1^ h^−1^, whereas the PRO group received 1–2 mg kg^−1^ of propofol bolus followed by a propofol infusion at 2–3 mg kg^−1^ h^−1^. During ERCP, the primary outcome was the incidence of hypoxemia (SpO_2_ < 90% for > 10 s). Other intraoperative adverse events were also recorded as secondary outcomes, including respiratory depression (respiratory rate of < 10 bpm min^−1^), hypotension (MAP < 65 mmHg), and bradycardia (HR < 45 beats min^−1^).

**Results:**

The incidence of hypoxemia was significantly reduced in the DEX group compared to the PRO group (0% versus 28.6%, respectively; *P* = 0.032). Patients in the PRO group exhibited respiratory depression more frequently than patients in the DEX group (35% versus 81%, respectively; *P* = 0.003). There were no significant differences in terms of hypotension and bradycardia episodes between groups. During the procedures, the satisfaction scores of endoscopists and patients, as well as the pain and procedure memory scores of patients were comparable between groups.

**Conclusion:**

In comparison with propofol, dexmedetomidine provided adequate sedation safety with no adverse effects on sedation efficacy during ERCP.

**Trial registration:**

Chinese Clinical Trial Registry, ChiCTR2200061468, 25/06/2022.

## Introduction

Endoscopic retrograde cholangiopancreatography (ERCP) is a complex procedure typically used for diagnostic or therapeutic purposes in pancreaticobiliary pathology while the patient is in a prone or semi-prone position. Sedation and anesthesia are usually required to relieve patient anxiety and discomfort, enhance examination outcomes, and diminish the patient’s memory of the procedure. It is notable that anesthesia care standards for ERCP have not been established and that sedation practice patterns vary substantially worldwide [1].

Many sedative medications, with various anesthetic mechanisms, are used to provide appropriate sedation and anesthesia levels for ERCP. In the past, ERCP was performed under moderate sedation using opioids and benzodiazepines, with or without adjunctive agents; however, moderate sedation has been largely abandoned due to insufficiency, resulting in premature completion [2, 3]. In recent decades, propofol or dexmedetomidine as adjuncts to moderate sedation have been considered [4, 5]. Due to its rapid onset of action and short half-life, propofol is used for painless endoscopy; however, it causes cardiovascular inhibition and dose-dependent respiratory depression, thereby necessitating constant monitoring by appropriately trained anesthetists [6, 7]. Furthermore, the prone or semi-prone position may be associated with altered cardiopulmonary physiology and restricted airway access during ERCP [8]. Therefore, airway management under propofol sedation may be further complicated and challenging in worst-case scenarios.

Dexmedetomidine is a highly selective α-2-adrenoceptor agonist that, at clinically deep sedation levels, provides analgesia and sedation with minimal cardiopulmonary compromise. Recent investigations have demonstrated that dexmedetomidine exerts synergistic effects when combined with opioids and benzodiazepines and provides adequate procedural sedation similar to propofol in various clinical scenarios^.^ [9–11] However, dexmedetomidine may induce bradycardia and sympathetic inhibition [12]. While both sedatives have their advantages and disadvantages, to the best of our knowledge, there is no consensus on the most effective, safe, and satisfying sedation regimen to facilitate ERCP procedures.

When considering previous evidence on dexmedetomidine efficacy and safety, we hypothesized that when compared with sedation by propofol, the drug combined with opioids and benzodiazepines could reduce the incidence of cardiopulmonary adverse events concomitant with equally satisfactory sedation conditions for the endoscopist and patient during ERCP procedures. Furthermore, midazolam is the benzodiazepine of preference for procedure memory effects that are fast-acting, reversible, and retrograde. Sufentanil has a high potency and affinity for the opioid receptor, but it has moderate respiratory depression. Hence, combined sufentanil and midazolam administration was used as the basic ERCP medication throughout our study.

We designed this study to compare the efficacy and safety of dexmedetomidine and propofol with balanced sufentanil and midazolam administration for sedation during ERCP procedures.

## Method

### Design and patients

This research was approved by the Medical Ethics Committee of the First Affiliated Hospital of Wenzhou Medical University and registered with the Clinical Trial Registration Center of China (ChiCTR2200061468, 25/06/2022). Patients who were 18 to 80 years old, had American Society of Anesthesiologists (ASA) physical status I–III, and were scheduled for an ERCP procedure between June 2022 and August 2022 were eligible to be enrolled in this prospective, randomized, single-blind study. Exclusion criteria included ASA physical status IV–V, refusal to participate, pregnant or breast-feeding patients, a history of allergy to study medication, and long-term sedative or narcotic analgesic drug abuse. All patients provided written informed consent.

### Anesthetic procedure

Patients fasted for a minimum of 6 h prior to ERCP. After intravenous access was obtained, an infusion of 500 mL of Ringer’s solution was initiated at a rate of 250 mL h^−1^. Patients were positioned in a semi-prone position, and 2 L min^−1^ of oxygen was administered through a nasal cannula. As premedication, patients were given laryngopharynx topical anesthesia with 2% lidocaine hydrochloride mucilage and 1% dyclonine hydrochloride mucilage, an intravenous dose of 0.2 μg kg^−1^ sufentanil, and 0.02 mg kg^−1^ midazolam. Additionally, the DEX group also received an initial bolus of 0.6 μg kg^−1^ dexmedetomidine over 2 min, followed by a dexmedetomidine infusion at 1.2 μg kg^−1^ h^−1^. The PRO group received an initial bolus of 1–2 mg kg^−1^ propofol over 30 s followed by a propofol infusion at 2–3 mg kg^−1^ h^−1^. We targeted a sedation level on the Ramsay Sedation Scale (RSS, Table 1) of ≥ 4. In case of RSS < 4 or intolerance to the procedure, the patients were administered 0.1 μg kg^−1^ sufentanil and 0.01 mg kg^−1^ midazolam as rescue drugs. Continuous infusion of propofol or dexmedetomidine were withheld if BIS values < 45.

**Table 1:** Ramsay sedation scale

Sedation score	Response
1	Anxious and agitated or restless, or both
2	Co-operative, oriented, and tranquil
3	Responding to commands only
4	Brisk response to light glabellar tap or loud auditory stimulus
5	Sluggish response to light glabellar tap or loud auditory stimulus
6	No response to stimulus

### Measurement

Age, gender, and body mass index were measured prior to surgery as baseline demographic data. The sedation level was evaluated using the RSS for clinical scoring and the bispectral index (BIS) as an objective tool. During the procedure, heart rate (HR), oxygen saturation (SpO_2_), respiratory rate (RR), mean blood pressure (MAP), RSS, and BIS levels were monitored and recorded at the following time points: 5 min before sedation (baseline, T_0_), 5 min after sedation (T_1_), 0, 5, 10, 15, and 20 min after starting ERCP (T_2_–T_6_), and post-procedure 0, 5, and 10 min (T_7_–T_9_). Patients were observed in the recovery unit for at least 30 min after the procedure. The recovery status was evaluated using the modified Aldrete Score [13]. Patients were ready for discharge when this score reached at least 9 without significant adverse effects such as nausea and dizziness.

Time to achieve RSS ≥ 4 was recorded as the onset time of targeted sedation. Recovery time was measured from the conclusion of ERCP until a modified Aldrete score of 9 was attained. Rescue drug injections were recorded. Immediately after the procedure, endoscopists were requested to assess their level of satisfaction: 1) satisfied, 2) moderately dissatisfied, 3) severely dissatisfied, and 4) unbearable. On the day following the procedure, the satisfaction score, pain, and procedure memory of the patients were evaluated by an anesthesia resident as follows: Satisfaction score: 1) comfortable, 2) mild discomfort, 3) severe discomfort, and 4) unbearable; there were four levels of pain: 1) no pain, 2) mild pain, 3) moderate pain, and 4) severe pain; Procedure memory: 1) I do not remember any part of the procedure; 2) I remember some parts of the procedure; and 3) I remember the entire procedure.

In terms of sedation regimen safety, hemodynamic and respiratory variables such as MAP, HR, SpO_2_, and RR were compared between groups. Sedation-related adverse events (SRAEs) and ERCP-related adverse events were among the perioperative adverse events. SRAEs were defined as hypoxemia (SpO_2_ < 90% for > 10 s), respiratory depression (RR < 10 bpm), hypotension (MAP < 65 mmHg), and bradycardia (HR < 45 beats min^−1^). Any sign of respiratory depression prompted an intervention which consisted of: 1) patient stimulation; 2) withholding medication; 3) a chin lift or jaw thrust maneuver; 4) increasing oxygen supplementation. If hypoxemia was observed, supplemental oxygen was administered through the nasopharyngeal airway. Intravenous saline or vasoactive agents (ephedrine or atropine) were used to treat hypotension or bradycardia. Post-ERCP pancreatitis (PEP), bleeding, perforation, and infection were categorized as ERCP-related adverse events. Intubation of the trachea and/or mechanical ventilation were considered severe adverse events.

### Outcomes

The primary outcome was the incidence of hypoxemia. The secondary outcomes included: (1) onset time of targeted sedation, recovery time, and rescue drug injections; (2) the satisfaction scores of endoscopists and patients, as well as the pain and procedure memory scores; (3) RSS, BIS, MAP, HR, SpO_2_, and RR at T_0_–T_9_; (4) adverse events recorded, which included respiratory depression, hypotension, bradycardia, and ERCP-related adverse events.

### Statistical analysis

We hypothesized that sedation with dexmedetomidine for ERCP would reduce the incidence of hypoxemia by at least 50% when compared to propofol. According to a previous study by Jokelainen et al. [14], 60.7% of patients who underwent ERCP under propofol-based sedation experienced hypoxemia. To achieve a power ≥ 0.80 and an α level = 0.05, a sample size of 17 patients per group was required for the chi-squared test with Fisher’s exact test. The final sample size was set at 19 patients per group, assuming a 10% dropout rate.

The SPSS software version 26.0 (IBM, Armonk, NY, USA) was used to conduct statistical analyses. An independent sample *t-*test and nonparametric tests were used for continuous variables. Pearson’s chi-squared and Fisher’s exact tests were applied to variables that were expressed as numbers and/or percentages of the total for categorical variables. Continuous variables with normal distributions are presented as the mean ± standard deviation. The Kolmogorov–Smirnov test was used to compare non-normally distributed data, which are presented as the median and interquartile range (IQR). A *P* value < 0.05 was considered statistically significant.

## Results

**Fig. 1:**
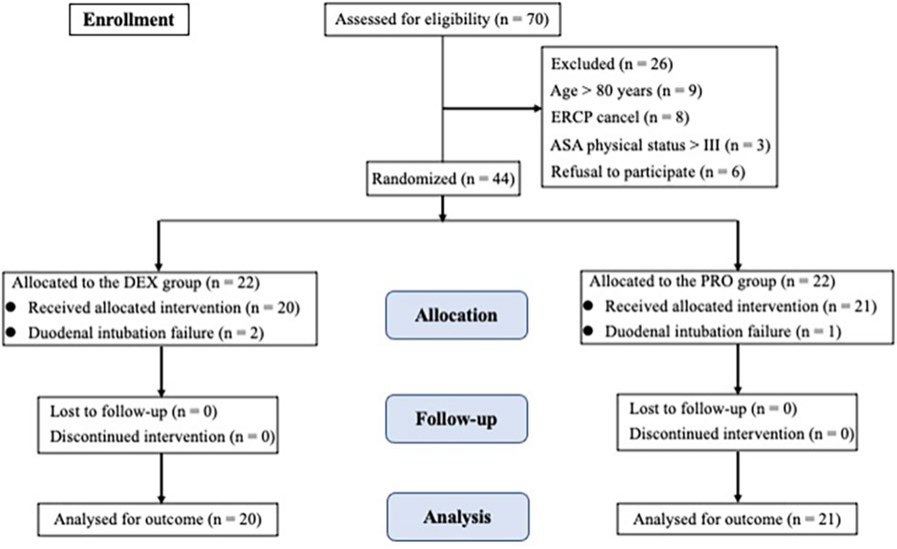
CONSORT flow diagram

In our gastrointestinal endoscopy unit, 44 eligible patients scheduled for an ERCP procedure were randomized, with 22 patients per group. Due to unsuccessful duodenal intubation, two patients from the DEX group and one from the PRO group were excluded. Therefore, data were ultimately collected from 20 and 21 patients in the DEX and PRO groups, respectively, as shown in the CONSORT flow diagram (Fig. 1).

The characteristics of patients and details of the ERCP procedure are shown in Table 2. In the DEX group, the average age of patients was 57, while in the PRO group, it was 61. Physical characteristics and underlying comorbidities were comparable between the two groups. Furthermore, no significant differences were identified between the groups in terms of indications for ERCP or the mean procedure duration (*P* > 0.05).

**Table 2:** Patient characteristics and ERCP procedure details

	DEX Group (*n* = 20)	PRO Group (*n* = 21)	*p* value
Age (y)	57.1 ± 16.1	61.0 ± 12.8	0.395
Sex (male/female, n)	9/11	9/12	0.890
BMI (kg/m^2^)	23.2 ± 2.6	22.7 ± 3.6	0.676
Cigarette smoking [n (%)]	4 (20.0)	1 (4.8)	0.311
Alcohol intake [n (%)]	3 (15.0)	5 (23.8)	0.477
Comorbidities [n (%)]			
Cardiovascular disease	6 (30.0)	9 (42.9)	0.393
Respiratory disease	None	4 (19.0)	0.126
Diabetes	3 (15.0)	5 (23.8)	0.751
Renal disease	6 (30.0)	2 (9.5)	0.208
Liver disease	7 (35.0)	12 (57.1)	0.155
ASA status (I/II/III, n)	4/10/6	2/16/3	0.284
ERCP indications [n (%)]			0.798
Common bile duct stone	17 (85.0)	16 (76.2)	
Biliary strictures	None	2 (9.5)	
Gallbladder carcinoma	1 (5.0)	None	
Pancreatic pathology	1 (5.0)	2 (9.5)	
Other	1 (5.0)	1 (4.8)	
Duration of procedures (min)	23.3 ± 11.5	25.5 ± 14.7	0.593

Normally distributed data are presented as mean ± SD; categorical variables are presented as count (%)

Abbreviations: *ERCP* Endoscopic retrograde cholangiopancreatography, *DEX* Dexmedetomidine, *PRO* Propofol, *BMI* Body mass index, *ASA* American Society of Anesthesiologists, *SD* Standard deviation

**Fig. 2:**
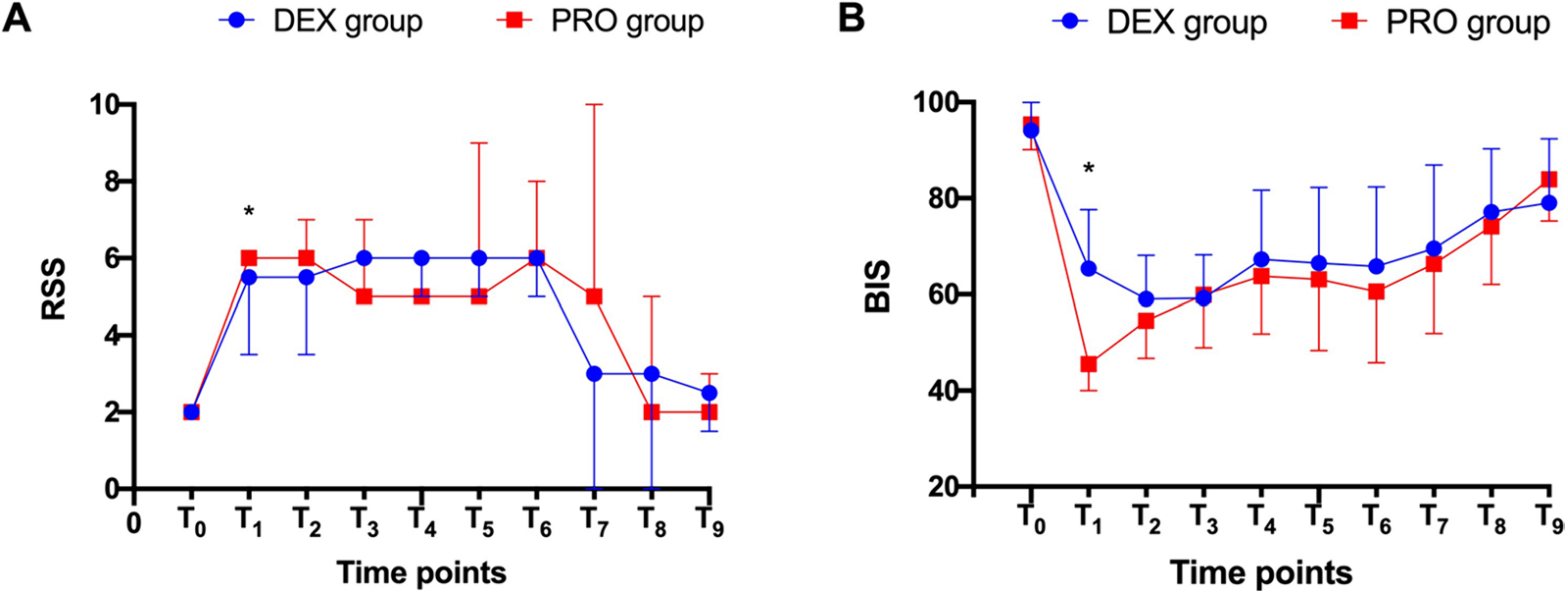
Comparison of RSS and BIS scores between the two groups. RSS, Ramsay sedation scale; BIS, bispectral index; ERCP, endoscopic retrograde cholangiopancreatography; DEX, dexmedetomidine; PRO, propofol. T_0_, 5 min before sedation; T_1_, 5 min after sedation; T_2_–T_6_, 0, 5, 10, 15, 20 min after starting ERCP; T_7_–T_9_, post-procedure 0, 5, 10 min

As indicated in Fig. 2, RSS and BIS scores were comparable at baseline, and no differences were observed at T_2_–T_9_ time points; however, at the T_1_ time point the RSS score was significantly higher while the BIS score was lower in the PRO group compared to the DEX group (*P* < 0.05).

Parameters of sedation efficacy are shown in Table 3. The PRO group demonstrated a quicker onset of targeted sedation compared to the DEX group. In contrast, patients in the DEX group required significantly less time to recuperate than those in the PRO group (*P* < 0.05). The requirements for administering rescue agents to facilitate procedures were similar between groups (*P* > 0.05).

**Table 3:** Parameters of sedation efficacy

	DEX Group (*n* = 20)	PRO Group (*n* = 21)	*p* value
Onset time of targeted sedation (min)	2.7 ± 1.8	1.0 ± 0.0	0.000
Recovery time (min)	1.6 ± 1.9	6.9 ± 3.2	0.002
Rescue drug injections [n (%)]	5 (25.0)	4 (19.0)	0.934

Normally distributed data are presented as mean ± SD; categorical variables are presented as count (%)

*Abbreviations*: *DEX* Dexmedetomidine, *PRO* Propofol, *SD* Standard deviation

**Fig. 3:**
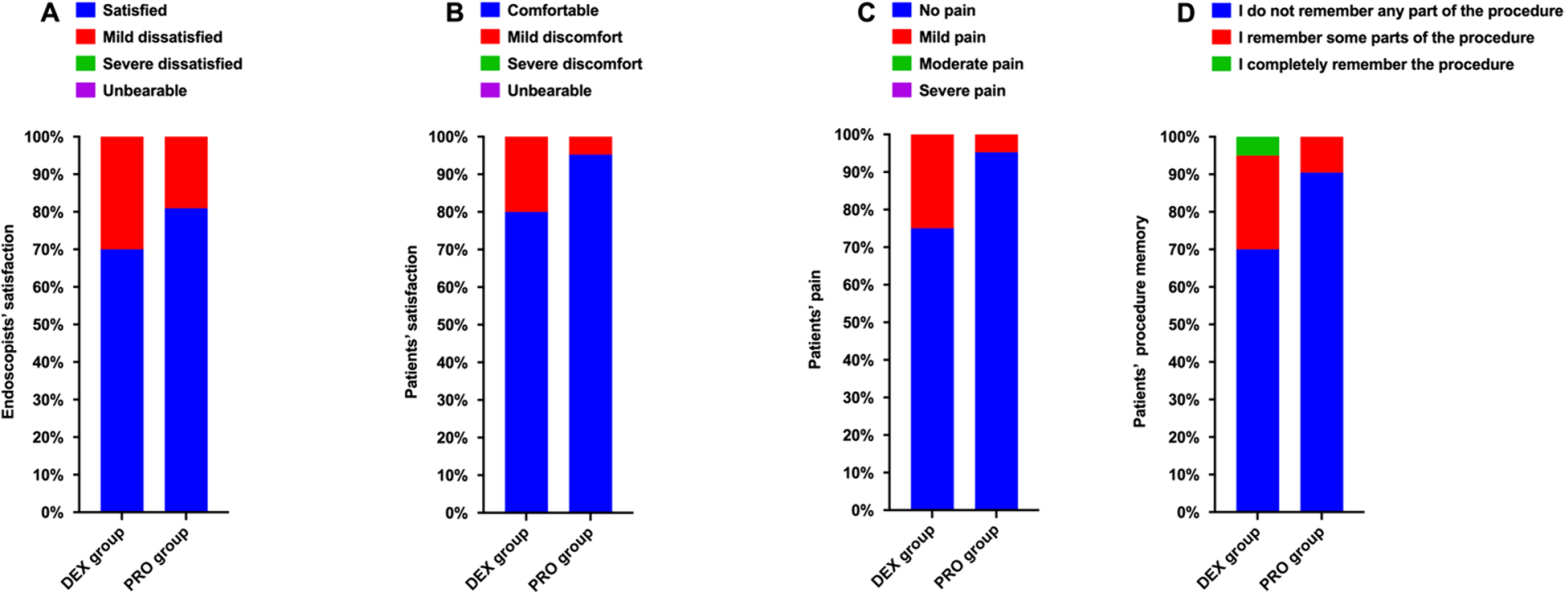
Comparison of the satisfaction score of endoscopists and patients, and pain and procedure memory scores of patients between the two groups. DEX, dexmedetomidine; PRO, propofol

Figure 3 depicts the satisfaction scores of the endoscopists and patients, as well as the pain and procedure memory scores of patients. The satisfaction scores of endoscopists and patient were comparable between groups, while no differences were identified between groups in terms of pain or procedure memory scores of patients (*P* > 0.05).

**Fig. 4:**
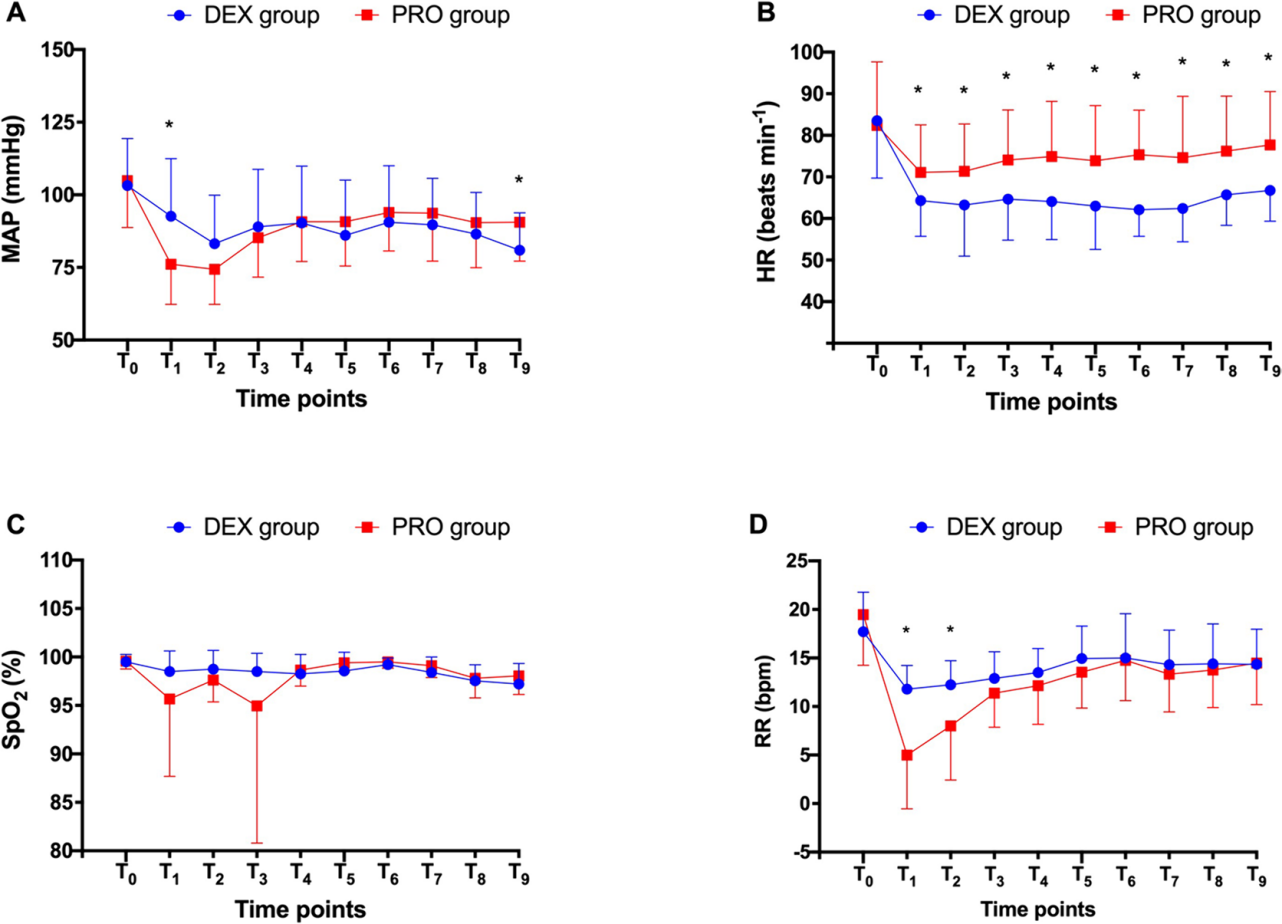
Comparison of MAP, HR, SpO_2_, and RR between the two groups. MAP, mean arterial pressure; HR, heart rate; SpO_2_, saturation of peripheral oxygen; RR, respiratory rate; DEX, dexmedetomidine; PRO, propofol. T_0_, 5 min before sedation; T_1_, 5 min after sedation; T_2_–T_6_, 0, 5, 10, 15, 20 min after starting ERCP; T_7_–T_9_, post-procedure 0, 5, 10 min

As shown in Fig. 4, there were no significant differences between groups in terms of MAP, HR, SpO_2_, and RR at T_0_ baseline. Patients in the DEX group exhibited a statistically significant higher MAP after the loading dose injection at T_1_, whereas patients in the PRO group exhibited a statistically significant higher MAP at T_9_ (*P* < 0.05). A statistical difference in HR was observed between the two groups from 5 min after the initiation of sedation until the conclusion of the procedure. Throughout the procedure, there was no difference in SpO_2_ between groups (*P* > 0.05), however, RR in the PRO group was significantly lower than in the DEX group at T_1_ and T_2_ (*P* < 0.05).

The SRAEs are indicated in Table 4. There were statistically significant differences in the incidence of hypoxemia and respiratory depression between groups (*P* < 0.05). Hypoxemia episodes occurred in six patients in the PRO group but none in the DEX group. Respiratory depression occurred in 81% of patients in the PRO group and 35% of patients in the DEX group (*P* < 0.05). In terms of hypotension and bradycardia episodes, there were no statistically significant differences between the groups (*P* > 0.05).

**Table 4:** Sedation-related adverse events

	DEX Group (*n *= 20)	PRO Group (*n *= 21)	*p* value
Hypoxemia [n (%)]	None	6 (28.6)	0.032
Respiratory depression [n (%)]	7 (35.0)	17 (81.0)	0.003
Hypotension [n (%)]	5 (25.0)	7 (33.3)	0.558
Bradycardia [n (%)]	5 (25.0)	2 (9.5)	0.367

The data are presented as count (%)

*Abbreviations: DEX* Dexmedetomidine, *PRO* Propofol

The adverse events related to ERCP are shown in Table 5, with no observed differences between groups. The length of hospital stay was similar between groups (*P* > 0.05). No severe adverse events occurred in either group.

**Table 5:** ERCP-related adverse events

	DEX Group (*n *= 20)	PRO Group (*n *= 21)	*p* value
PEP [n (%)]	5 (25.0)	4 (19.1)	0.934
Bleeding [n (%)]	None	1 (4.8)	>0.999
Perforation [n (%)]	None	None	1.000
Infection [n (%)]	6 (30.0)	4 (19.1)	0.651
Length of hospital stay (day)	7.7 ± 4.7	6.5 ± 4.7	0.579

Normally distributed data are presented as mean ± SD; categorical variables are presented as count (%)

*Abbreviations: ERCP* Endoscopic retrograde cholangiopancreatography, *PEP* Post-endoscopic retrograde cholangiopancreatography pancreatitis, *DEX* Dexmedetomidine, *PRO* Propofol

## Discussion

Based on the results of this study, a dexmedetomidine-based sedation regimen provided a superior level of safety, with minimal respiratory depression during ERCP and no downstream effects on procedural efficacy when compared with propofol sedation.

ERCP is an essential diagnostic and therapeutic procedure for biliary and pancreatic diseases and is generally conducted under anesthesia due to its painful and time-consuming properties. Sedation without intubation administered by an anesthesiologist appears to be the most recommended technique for ERCP due to a reduced incidence of adverse events [15]. Propofol is the most commonly used sedative for sedation without intubation during gastrointestinal endoscopy due to its potent action, rapid onset, and fast recovery [16]. However, doubts concerning its sedation-related side effects hinder its widespread clinical application, particularly during ERCP for elderly populations and those requiring prone positions. Furthermore, previous studies have reported a high incidence of hypotension (4.8%–19%) and hypoxemia or apnea (3.3%–60.7%) during ERCP under propofol sedation [6, 14, 17, 18]. Dexmedetomidine is a highly selective α-2-adrenoceptor agonist with sedative, analgesic, and anxiolytic properties. Balanced dexmedetomidine administration with opioids and benzodiazepines could be a safer alternative to propofol during advanced endoscopic procedures. However, to our knowledge, there is limited data on the sedation efficacy and safety of dexmedetomidine combined with opioids and benzodiazepine administration when compared with propofol administration during ERCP.

During ERCP procedures, the sedation efficacy of dexmedetomidine combined with opioids and benzodiazepines was investigated in several controlled, randomized trials [4, 19]. Lu et al. [4] demonstrated that 95% of patients under dexmedetomidine and remifentanil anesthesia underwent ERCP without discomfort or additional sedative use. Ikeda et al. [19] showed that combination treatment with dexmedetomidine and benzodiazepines provided high-quality sedative effects with rare excessive movements. Consistent with previous results, all the patients in our study achieved the targeted RSS sedation level and underwent the entire procedure without premature termination. In addition, RSS sedation levels and BIS scores were comparable between groups after bolus administration, with no significant differences in the need for rescue agents.

Patients undergoing gastrointestinal endoscopy were more satisfied with propofol than with dexmedetomidine, according to a previous meta-analysis [20], whereas endoscopists were more satisfied with dexmedetomidine-based sedation than with propofol, according to another meta-analysis [21]. According to the results of the present study, different anesthetic agents produced comparable satisfaction levels among endoscopists and patients. Such a discrepancy may be attributed to the synergistic effects of opioids and benzodiazepines when added to dexmedetomidine sedation [10]. Additionally, the effect of suppressing gastric motility with dexmedetomidine-based sedation may contribute to enhanced endoscopist satisfaction when compared with propofol [11]. In addition, our research revealed that both sedative regimens provided equivalent sedation efficacy with relatively satisfactory levels of sedation.

It is worth noting that the sedation depth was transiently deeper at T_1_ in the PRO group compared to the DEX group. This was a result of the rapid onset of propofol activity, which was confirmed by the reduced onset time of targeted sedation in the PRO group compared to the DEX group. This may be a double-edged sword in terms of shorter onset times for sedation. As shown in Fig. 4, MAP and RR were significantly lower at T_1_ in the PRO group. This finding reflected the narrow therapeutic index of propofol and the risk of cardiovascular complications, particularly during the induction period. This indicated a difficulty in controlling cardiorespiratory stability with propofol, particularly in elderly patients, by an inexperienced anesthesiologist or under nurse-guided sedation [22]. In contrast, patients in the DEX group recovered faster than those in the PRO group due to the unique property of its arousable sedation [10]. Immediately after ERCP, patients in the DEX group were aroused by their name being called or being gently shaken. In addition, dexmedetomidine enabled patients to achieve an Aldrete score of 9 at the end of the procedure and to leave the operation room within two minutes.

Respiratory and cardiovascular depression are well known as the adverse effects of propofol. During sedation, the features that differentiate dexmedetomidine from propofol are the maintenance of spontaneous breathing and the avoidance of profound cardiovascular compromise. In our study, MAP was lower after induction but higher during the recovery period under propofol sedation when compared with dexmedetomidine. The short elimination half-life of propofol may be responsible for these differences, whereas dexmedetomidine has a relatively slow elimination with cumulative effects leading to prolonged cardiovascular depression [23]. Consequently, dexmedetomidine and propofol may present comparable hypotension risks during the procedure. Respiratory depression dependent on dose is another significant concern with propofol treatment [6]. In our study, 29% of patients required airway manipulation due to transient hypoxemia, while 81% of cases exhibited a respiratory rate < 10 bpm in the PRO group. Although no significant differences in SpO_2_ were observed between the two segments, fluctuations in values were obvious under propofol sedation. These results agreed with those of Yang et al. [24] who showed that 28% of patients experienced hypoxemia during propofol sedation. The major side effect of dexmedetomidine is bradycardia resulting from α 2-adrenoceptor activation; we observed a lower heart rate in the DEX group throughout the entire study, which could be immediately reversed by treatment with atropine.

In recent years, several case reports have suggested an association between short-term exposure to propofol and acute pancreatitis [25, 26]. Therefore propofol sedation has raised additional concerns about increasing the risk of post-ERCP pancreatitis (PEP). Consistent with the findings of Li et al. [27], our ERCP procedure lasted for 25 min on average, and our results demonstrated that such a short-term exposure to propofol exhibited a similar risk to dexmedetomidine in terms of PEP. Furthermore, previous investigations indicated that dexmedetomidine may provide superior anti-inflammatory effects and reduce the risk of infection [28]. However, no significant differences in the incidence of post-ERCP infections were observed between groups. The length of hospital stay was not influenced by sedating regimens in this study, which differed from the findings by Zhang et al. [29] This discrepancy may be attributed to the similar rates of ERCP-related adverse events. Based on these data, propofol or dexmedetomidine sedation are comparatively safe anesthetic strategies for ERCP that do not increase the risk of ERCP-related adverse events.

The standard recommendation for dexmedetomidine administration method is complicated, as the loading dose should be given over 10 min, followed by an infusion rate of 0.7–1.4 μg kg^−1^ h^−1^ [12] However, data from previous studies demonstrate that 0.5 μg kg^−1^ dexmedetomidine could be administered as a bolus within 30 s in patients without causing any significant hemodynamic compromise [18, 30]. Therefore, in our study, we used 0.6 μg kg^−1^ loading doses of dexmedetomidine over 2 min, alternated with sufentanil and midazolam.

Our research had several limitations. Patients with ASA status I–III and < 80 years old were included in our single-center study. Age and an ASA status of III or higher were demonstrated to be independent predictors of the development of SRAEs during propofol sedation [24]. Additionally, the beneficial effects of dexmedetomidine-based sedation may have been more pronounced in critically ill patients; this factor may have influenced our results.

## Conclusion

Both dexmedetomidine and propofol-based sedating regimens exhibited adequate sedative efficacy for ERCP. Dexmedetomidine, in combination with opioids and benzodiazepines, offered distinct advantages in terms of sedation safety due to a lower incidence of adverse respiratory events during ERCP when compared with propofol. Therefore, dexmedetomidine may be an appropriate alternative to propofol for patients undergoing ERCP.

## Data Availability

The datasets used and/or analysed during the current study available from the corresponding author on reasonable request.

## Abbreviations

ERCP: Endoscopic retrograde cholangiopancreatography
SRAEs: Sedation-related adverse events
ASA: American Society of Anesthesiologists
RSS: Ramsay Sedation Scale
BIS: Bispectral index
HR: Heart rate
SpO_2_: Oxygen saturation
RR: Respiratory rate
MAP: Mean blood pressure
PEP: Post-ERCP pancreatitis
IQR: Interquartile range
